# Causes of death in very preterm infants cared for in neonatal intensive care units: a population-based retrospective cohort study

**DOI:** 10.1186/s12887-017-0810-3

**Published:** 2017-02-21

**Authors:** Tim Schindler, Louise Koller-Smith, Kei Lui, Barbara Bajuk, Srinivas Bolisetty, New South Wales and Australian Capital Territory Neonatal Intensive Care Units’ Data Collection

**Affiliations:** 1 Faculty of Medicine, University of New South Wales, Sydney, Australia; 2 Department of Newborn Care, Royal Hospital for Women, Sydney, Australia; 3 New South Wales Pregnancy and Newborn Services Network (PSN), Sydney, Australia

**Keywords:** Preterm, Infant, Mortality, Cause of death

## Abstract

**Background:**

While there are good data to describe changing trends in mortality and morbidity rates for preterm populations, there is very little information on the specific causes and pattern of death in terms of age of vulnerability. It is well established that mortality increases with decreasing gestational age but there are limited data on the specific causes that account for this increased mortality. The aim of this study was to establish the common causes of hospital mortality in a regional preterm population admitted to a neonatal intensive care unit (NICU).

**Methods:**

Retrospective analysis of prospectively collected data of the Neonatal Intensive Care Units' (NICUS) Data Collection of all 10 NICUs in the region. Infants <32 weeks gestation without major congenital anomalies admitted from 2007 to 2011 were included. Three authors reviewed all cases to agree upon the immediate cause of death.

**Results:**

There were 345 (7.7%) deaths out of 4454 infants. The most common cause of death across all gestational groups was major IVH (cause-specific mortality rate [CMR] 22 per 1000 infants), followed by acute respiratory illnesses [ARI] (CMR 21 per 1000 infants) and sepsis (CMR 12 per 1000 infants). The most common cause of death was different in each gestational group (22–25 weeks [ARI], 26–28 weeks [IVH] and 29–31 weeks [perinatal asphyxia]). Pregnancy induced hypertension, antenatal steroids and chorioamnionitis were all associated with changes in CMRs. Deaths due to ARI or major IVH were more likely to occur at an earlier age (median [quartiles] 1.4 [0.3–4.4] and 3.6 [1.9–6.6] days respectively) in comparison to NEC and miscellaneous causes (25.2 [15.4–37.3] and 25.8 [3.2–68.9] days respectively).

**Conclusions:**

Major IVH and ARI were the most common causes of hospital mortality in this extreme to very preterm population. Perinatal factors have a significant impact on cause-specific mortality. The varying timing of death provides insight into the prolonged vulnerability for diseases such as necrotising enterocolitis in our preterm population.

## Background

For many parents, premature birth is a confronting experience whereby they are faced with a series of potentially life-changing events as their baby transitions from foetal to neonatal life. As clinicians, it is important to have a thorough understanding of preterm mortality as we counsel and prognosticate through this process. While there are good data to describe changing trends in mortality and morbidity rates for preterm populations [1–3], there is very little information on the specific causes and pattern of death in terms of age of vulnerability. It is well established that mortality increases with decreasing gestational age but there are limited data on the specific causes that account for this increased mortality.

Cause-specific mortality rates (CMR) are routinely used to describe the mortality rate from a specified cause for a population in a defined period. There are studies that addressed the cause-specific mortality in the neonatal intensive care environment and the newborn period, however, these studies generally accepted extreme prematurity or immaturity as a cause of death without specifying the immediate cause of death for this population [4–7].

We have previously published mortality data on a large cohort of preterm infants less than 32 weeks gestation admitted to a regional network of neonatal intensive care units [8]. We reported gestational age-specific mortality, including identification of perinatal risk factors that have a significant impact on overall mortality. In this study, we performed an in-depth analysis of the immediate causes of mortality in this same cohort. We investigated the CMR by gestational age at birth. We also investigated the age at death based on the cause of mortality. We also analysed the implications of the cause specific mortality on resource utilisation.

## Methods

Data for this study were sourced from the prospectively collected Neonatal Intensive Care Units’ (NICUS) Data Collection. NICUS Data Collection is an ongoing regional audit of neonates admitted to all 10 NICUs in New South Wales (NSW) and Australian Capital Territory (ACT). All 10 NICUs are level III or level IV facilities [9]. These units are regularly benchmarked against each other and have comparable outcomes with respect to mortality and major morbidity. Neonatal, maternal and perinatal data are prospectively collected within each NICU by a designated Clinical Audit Officer. The data undergo rigorous quality control procedures with regular audit and validation checks. All liveborn infants less than 32 weeks gestation admitted to any of these NICUs from 1^st^ January 2007 to 31st December 2011 were identified. Infants with major congenital malformations (constituting major confounding factors for morbidity and mortality) or admitted for palliation were excluded from the analysis. The perinatal characteristics of these infants, along with gestational age-specific hospital survival rates, hospital morbidities and interventions are detailed in our earlier study [8]. This a population where the majority of women received antenatal care (97.7%), antenatal steroids (90%) and delivered at a tertiary perinatal centre (89%).

Study variables were defined according to NICUS Data Collection Manual. Definitions for other relevant major outcomes are as follows:

*Cause-specific mortality rate:* Number of deaths prior to hospital discharge from a specified cause for every 1000 infants admitted to a NICU. For instance, CMR for major IVH was calculated as per the formula below:

$$ \frac{\mathrm{Number}\kern0.2em \mathrm{of}\kern0.2em \mathrm{deaths}\kern0.2em \mathrm{due}\kern0.2em \mathrm{to}\kern0.2em \mathrm{Major}\kern0.2em \mathrm{IVH}\kern0.2em \mathrm{x}\kern0.2em 1000}{\mathrm{Study}\kern0.2em \mathrm{cohort}} $$

*Ventilation days*: Respiratory support with either mechanical ventilation, CPAP or high flow support (≥2 liters per minute).

*Immediate cause of death:* The disease, injury or complication that directly preceded death. Three authors *(TS, SB and BB)* reviewed all cases of hospital death that occurred before first discharge from hospital to home. This does not include deaths that may have occurred after hospital discharge. The cause of death recorded in the NICUS Data Collection was compared to the NICUS discharge summary for all cases. Each case was discussed in detail by all authors in each case. In cases where the immediate cause of death was unclear, further information from the case notes was sought from the individual units. For example, there were 48 cases where extreme prematurity was labelled as the immediate cause of death and these cases were further investigated to determine the complication that directly preceded death. If the immediate cause of death remained unclear at this point or if a consensus between the authors could not be reached, the individual unit was contacted directly to clarify the immediate cause of death. There were a number of cases where more than one problem could have been responsible for death. In these cases, the most significant problem, determined by the individual unit, was taken as the cause of death. In cases where intensive care was withdrawn, the antecedent problem leading to redirection of care was taken as the cause of death. In all cases, the authors were able to come to a consensus agreement on the immediate cause of death.

Deaths were categorised into the five most common groups and a miscellaneous group as defined below:

*Deaths due to acute respiratory illness:* Death from acute respiratory problems including hyaline membrane disease, pulmonary hypertension, pulmonary haemorrhage and pulmonary hypoplasia. Deaths attributable to chronic lung disease were not included in this group.

*Deaths due to sepsis:* Deaths attributed to either early or late onset sepsis. This included deaths that occurred in either the acute or subacute phase of illness. For example, an infant that progressed to subsequent organ failure as a result of sepsis would be classified as a death due to sepsis. These deaths were not differentiated by causative organism.

*Deaths due to intraventricular haemorrhage:* Any death attributable to Grade 3 or higher intraventricular and/or intracerebral haemorrhage. This included deaths following redirection of care because of a major haemorrhage.

*Deaths due to perinatal asphyxia:* Deaths that the individual unit attributed to any perinatal hypoxic insult. This included deaths that occurred in either the acute or subacute phase of illness. For example, an infant that progressed to subsequent organ failure as a result of an asphyxic event would be classified as a death due to perinatal asphyxia.

*Deaths due to necrotising enterocolitis:* Deaths directly attributable to the development of necrotising enterocolitis. This included deaths that occurred in either the acute or subacute phase of illness.

*Deaths due to miscellaneous causes:* All remaining deaths attributable to any cause not included in the five most common groups. Deaths attributable to chronic lung disease were included in this group.

These five most common groups and the remaining group of deaths due to miscellaneous causes were further analysed to identify the underlying perinatal risk factors and the pattern of death, including age at death and resource utilisation. Resource utilisation was measured by hours for respiratory support and parenteral nutrition and days for length of level 3 stay.

Statistical analyses were performed using SPSS Predictive Analytics Software (version 21, Chicago, Illinois, USA). Results were summarized as proportions, mean (±SD) and median and quartiles; Chi-square, parametric or non-parametric analyses were used where appropriate. Logistic multivariate analyses were used to determine independent significant factors associated with mortality. A Kaplan-Meier plot was created to illustrate the different chronological ages of death according to the cause of death. The study was approved by the South Eastern Sydney Illawarra Area Health Services Northern Hospital Network Human Research Ethics Committee.

## Results

During the study period, there were 4501 eligible neonates registered in the NICUS database. There were 44 neonates with major congenital malformations, two born at 22 weeks gestation and one at 24 weeks gestation admitted to NICU for palliation and they were excluded from the study. Of 4454 infants included, there were 345 deaths during the study period.

Table 1 shows the results of the univariate and multivariate analyses on perinatal characteristics between infants who survived and those who died prior to discharge. Extreme prematurity was strongly associated with increased mortality. Using 29–31 weeks gestation as referent, 26–28 week infants and 22–25 week infants had adjusted odds ratios (95% CI) of 3.46 (2.24–5.35) and 16.34 (10.23–25.97) respectively. Other perinatal factors associated with increased mortality were male gender, small for gestation and low Apgar score. Neonatal factors associated with increased mortality were early onset sepsis, persistent pulmonary hypertension, pulmonary haemorrhage, major IVH, moderate-severe hypoxic ischaemic encephalopathy and NEC. Breech delivery was associated with mortality but this was not significant after adjustment for risk factors. Similarly, the increased mortality associated with surfactant treated hyaline membrane disease was not significant after multivariate analysis.

**Table 1:** Perinatal and clinical characteristics of infants who died

Variable	Survived	Died	Univariate analysis	P	Multivariate analysis
	*n = 4109*	*n = 345*	OR (95% CI)		Adjusted OR (95% CI)
Maternal age(mean ± SD)	30 ± 6	30 ± 6	---	0.978	---
Maternal indigenous status	229 (6%)	22 (6%)	1.15 (0.73–1.81)	0.301	---
Attended antenatal care	4021 (98%)	330 (96%)	0.48 (0.27–0.84)	0.009	0.83 (0.39–1.77)
Assisted conception	512 (13%)	41 (12%)	0.94 (0.67–1.33)	0.761	---
Multiple Births	1210 (29%)	82 (24%)	0.75 (0.58–0.97)	0.026	1.03 (0.72–1.47)
Hypertensive Disease of Pregnancy	802 (20%)	46 (13%)	0.63 (0.46–0.87)	0.005	0.93 (0.59–1.45)
Antepartum Haemorrhage	985 (24%)	123 (36%)	1.76 (1.39–2.21)	0.000	1.23 (0.90–1.68)
Chorioamnionitis	838 (20%)	94 (27%)	1.46 (1.14–1.87)	0.003	0.89 (0.76–1.04)
Antenatal Steroids	3724 (91%)	288 (84%)	0.52 (0.38–0.71)	0.000	0.84 (0.53–1.32)
Out born	452 (11%)	52 (15%)	1.44 (1.05–1.96)	0.022	0.98 (0.62–1.56)
Vaginal Breech Delivery	210 (5%)	62 (18%)	4.07 (2.99–5.54)	0.000	1.33 (0.87–2.04)
Gestation, weeks	29 ± 2	26 ± 2	---	0.000	---
Gestation					
22–25 weeks	344	178	---	0.000	16.34 (10.23–25.97)
4026–28 weeks	1248	118			3.46 (2.24–5.35)
4029–31 weeks	2517	49			1.00 (referent)
Male gender	2134 (52%)	223 (65%)	1.69 (1.34–2.13)	0.000	1.47 (1.10–1.96)
Birth weight, g	1285 ± 377	895 ± 362	---	0.000	---
Small for Gestational Age	286 (7%)	37 (11%)	1.61 (1.12–2.31)	0.009	2.14 (1.28–3.56)
APGAR <7 at 5 mins	697 (17%)	186 (56%)	5.73 (4.56–7.18)	0.000	2.41 (1.79–3.23)
Early Onset Sepsis	66 (2%)	28 (8%)	5.41 (3.43–8.54)	0.000	4.21 (2.34–7.56)
Late Onset Sepsis	742 (18%)	75 (22%)	1.26 (0.96–1.65)	0.054	---
Surfactant treated HMD	2371 (58%)	294 (85%)	4.23 (3.12–5.73)	0.000	0.84 (0.55–1.27)
PPHN	143 (3%)	62 (18%)	6.08 (4.41–8.38)	0.000	3.06 (2.02–4.64)
Pulmonary haemorrhage	69 (2%)	58 (17%)	11.8 (8.18–17.1)	0.000	4.26 (2.65–6.87)
IVH Grade III/IV	96 (2%)	112 (32%)	20.1 (14.8–27.2)	0.000	9.27 (6.30–13.64)
HIE Stage 2	8 (0.2%)	24 (7%)	38.3 (17.0–86.0)	0.000	4.52 (3.18–6.43)
NEC Stage 2	92 (2%)	40 (12%)	5.73 (3.88–8.45)	0.000	2.02 (1.59–2.57)

The number of infants n (%), mean ± SD and median (quartiles) are shown. Significant variables defined by univariate analysis are entered into a multivariate analysis. Odds ratio and adjusted odds ratios are shown

The cause-specific mortality patterns based on individual perinatal factors are represented in Table 2. In comparison to infants who survived, infants who died from sepsis were more likely to have a history of chorioamnionitis. Infants who died from NEC were more likely to be small for gestational age, had younger mothers, were more likely to have a history of hypertensive disease during pregnancy and less likely to have a history of chorioamnionitis. Infants who died due to major IVH were less likely to have a history of hypertensive disease during pregnancy, more likely to have a history of antepartum haemorrhage, less likely to have received antenatal steroids and more likely to be outborn. With respect to ARI, infants were less likely to have received antenatal steroids and more likely to have a history of antepartum haemorrhage. Infants who died due to asphyxia had older mothers, were less likely to have had antenatal care or receive antenatal steroids and were more likely to have a history of antepartum haemorrhage. The gestational ages and birthweights of infants who died due to asphyxia were higher than those who died from other causes.

**Table 2:** Perinatal characteristics based on the cause-specific mortality

		Causes of death									
Variable	Survived	Sepsis		Necrotizing enterocolitis		Major IVH		Respiratory		Perinatal asphyxia	
*Total*	*4109*	*55*	P	*39*	P	*99*	P	*95*	P	*25*	P
Maternal Age	30 ± 6	31 ± 5	0.326	28 ± 6	0.049	30 ± 6	0.719	30 ± 7	0.413	33 ± 5	0.029
Caucasian	3218 (78%)	44 (80%)	0.763	33 (85%)	0.342	77 (78%)	0.898	77 (81%)	0.522	22 (88%)	0.241
Antenatal Care	4021 (98%)	52 (95%)	0.095	37 (95%)	0.203	95 (96%)	0.202	92 (97%)	0.501	23 (92%)	0.045
Assisted Conception	512 (13%)	10 (18%)	0.203	7 (18%)	0.303	11 (11%)	0.688	11 (12%)	0.797	1 (4%)	0.201
Multiple											
Twins	1084 (26%)	14 (26%)	0.877	9 (23%)	0.641	19 (19%)	0.108	21 (22%)	0.349	3 (12%)	0.103
Triplets	115 (3%)	2 (4%)	0.709	2 (5%)	0.382	2 (2%)	0.642	3 (3%)	0.834	2 (8%)	0.118
Hypertensive Disease of Pregnancy	802 (20%)	10 (18.2%)	0.804	12 (30.8%)	0.078	6 (6.1%)	0.001	11 (11.6%)	0.053	5 (20%)	0.952
Antepartum Haemorrhage	985 (24%)	15 (27.3%)	0.569	13 (33.3%)	0.173	33 (33.3%)	0.032	36 (37.9%)	0.002	14 (56%)	0.000
Chorioamnionitis	838 (20%)	28 (51%)	0.000	3 (8%)	0.050	21 (21%)	0.842	26 (27%)	0.096	6 (24%)	0.656
No Antenatal Steroids	385 (9%)	2 (4%)	0.146	3 (8%)	0.720	23 (23%)	0.000	22 (23%)	0.000	7 (28%)	0.002
Out born	452 (11%)	5 (9%)	0.653	2 (5%)	0.242	28 (28%)	0.000	12 (13%)	0.616	1 (4%)	0.264
Mode of Delivery											
Normal Vaginal	1253 (31%)	13 (24%)	0.272	10 (26%)	0.512	35 (35%)	0.300	25 (26%)	0.381	3 (12%)	0.045
Vaginal Breech	210 (5%)	12 (22%)	0.000	1 (3%)	0.471	19 (19%)	0.000	21 (22%)	0.000	4 (16%)	0.014
Instrumental	79 (2%)	1 (1.8%)	0.955	0 (0%)	0.382	2 (2%)	0.944	0 (0%)	0.172	0 (0%)	0.484
LSCS	2562 (62%)	29 (53%)	0.144	28 (72%)	0.225	43 (43%)	0.000	49 (52%)	0.032	18 (72%)	0.321
Gestation, weeks	29 ± 2	26 ± 2	0.000	27 ± 2	0.000	25 ± 2	0.000	25 ± 2	0.000	29 ± 2	0.456
Male gender	2134 (52%)	28 (51%)	0.880	27 (69%)	0.031	69 (70%)	0.000	61 (64%)	0.018	18 (72%)	0.045
Birth weight, g	1285 ± 377	865 ± 289	0.000	895 ± 243	0.000	836 ± 225	0.000	807 ± 280	0.000	1343 ± 478	0.446
Birth weight <10^th^ percentile	508 (12%)	9 (16%)	0.371	9 (23%)	0.044	9 (9%)	0.327	10 (11%)	0.590	4 (16%)	0.582
APGAR <7 at 5 mins	697 (17%)	26 (47%)	0.000	8 (21%)	0.566	58 (59%)	0.000	63 (69%)	0.000	22 (88%)	0.000
Resources utilisation											
Length of NICU Stay, days	56.8 (41.9–78.7)	7.3 (1.0–18.1)	0.000	25.2 (15.4–37.3)	0.000	3.6 (1.9–6.6)	0.000	1.4 (0.3–4.4)	0.000	2.5 (0.5–3.5)	0.000
Duration of Resp Support, hours	194 (40–853)	173 (13–421)	0.020	457 (267–785)	0.002	86 (44–145)	0.000	31 (7–104)	0.000	57 (12–84)	0.001
Duration of TPN, hours	231 (134–387)	143 (5–417)	0.004	374 (247–635)	0.000	42 (0–109)	0.000	0 (0–60)	0.000	0 (0–48)	0.000

*n* (%), (mean ± SD) and median (quartiles) are shown, Chi-square, *t*-test and Mann–Whitney U tests are used where appropriate. Perinatal characteristics for each cause of death are compared characteristics of survivors

Table 3 shows the distribution of the common causes of death stratified by gestation. The most common cause of death across all gestational groups was major IVH (CMR 22 per 1000 infants), followed by an acute respiratory illness [ARI] (CMR 21 per 1000 infants) and sepsis (CMR 12 per 1000 infants). In infants born at 22–25 weeks gestation, the commonest cause of death was ARI (CMR 119 per 1000 infants), followed by IVH (CMR 117 per 1000 infants) and sepsis (CMR 54 per 1000 infants). Chronic lung disease had a CMR of 13 per 1000 infants in this group. In infants born at 26–28 weeks gestation, the commonest cause of death was IVH (CMR 24 per 1000 infants), while infants born at 29–31 weeks gestation, it was perinatal asphyxia (CMR 5 per 1000 infants).

**Table 3:** Causes of death stratified by gestational age

	Gestational age group			
Cause of death	22–25 weeks	26–28 weeks	29–31 weeks	All GA groups
Total number of neonates	*n = 522*	*n = 1366*	*n = 2566*	*n = 4454*
Total deaths	178 (341/1000)	118 (86/1000)	49 (19/1000)	345 (77/1000)
Sepsis	28 (54/1000)	22 (16/1000)	5 (2/1000)	55 (12/1000)
NEC	9 (17/1000)	23 (17/1000)	7 (3/1000)	39 (9/1000)
Major IVH	61 (117/1000)	33 (24/1000)	5 (2/1000)	99 (22/1000)
Respiratory	62 (119/1000)	23 (17/1000)	10 (4/1000)	95 (21/1000)
HMD	47 (90/1000)	8 (6/1000)	0 (0/1000)	55 (12/1000)
Pulm HTN	2 (4/1000)	4 (3/1000)	2 (1/1000)	8 (2/1000)
Pulm Haem	6 (11/1000)	4 (3/1000)	2 (1/1000)	12 (3/1000)
Pulm Hypopl	7 (13/1000)	6 (4/1000)	6 (2/1000)	19 (4/1000)
PTX	0 (0/1000)	1 (1/1000)	0 (0/1000)	1 (0/1000)
Perinatal Asphyxia	2 (4/1000)	9 (7/1000)	14 (5/1000)	25 (6/1000)
Miscellaneous	16 (31/1000)	8 (6/1000)	8 (3/1000)	32 (7/1000)
CLD	7 (13/1000)	1 (1/1000)	1 (<1/1000)	9 (2/1000)

The number in parenthesis represents the cause-specific mortality rate (CMR) per 1000 infants

**Fig. 1:**
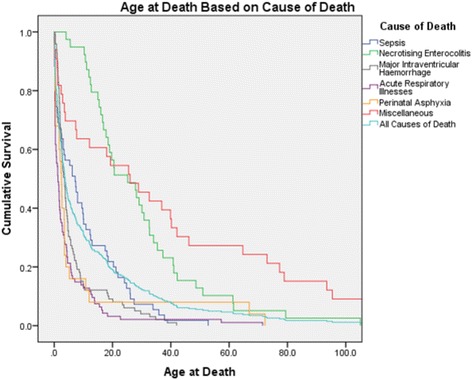
Kaplan-Meier plot illustrating the cause specific mortality according to the age of death

A Kaplan-Meier curve (Fig. 1) was created to illustrate the age of death based on the most common causes of death. The overall survival curve shows that 80% of infants who die, die within the first three weeks and 90% die within the first five weeks of life. Cause-specific curves demonstrate that infants who die due to ARI or major IVH are more likely to die early (median age [quartiles] at death 1.4 [0.3–4.4] and 3.6 [1.9–6.6] days respectively). Infants who die from NEC and miscellaneous causes are more likely to die later (median age at death 25.2 [15.4–37.3] and 25.8 [3.2–68.9] days respectively).

**Fig. 2:**
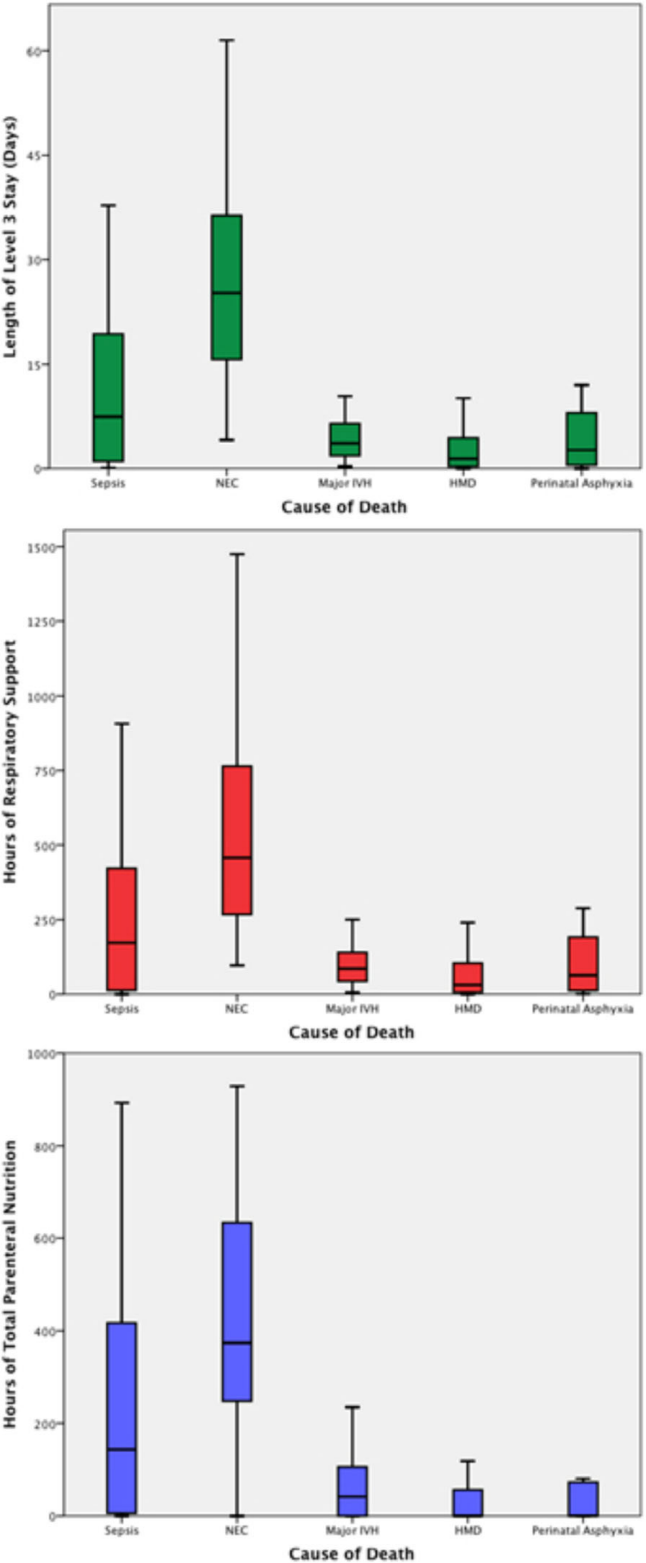
Comparison of resource utilisation from birth in relation to cause of death category: Top – length of level 3 stay (days); Middle – hours of respiratory support; Bottom – hours of parenteral nutrition. Median (*bar*), quartiles (*box*) and 95% CI (error bars) are shown

Resource utilisation from birth is compared for each of the most common causes of death in Fig. 2. Length of level 3 stay in days, hours of respiratory support and hours of parenteral nutrition have been used as surrogate markers for resource utilisation. Resource utilisation is highest in infants who die of NEC for length of level 3 stay, duration of respiratory support and duration of parenteral nutrition and lowest in infants who die from respiratory problems.

## Discussion

To our knowledge, this is the largest population based study describing CMR for an extreme to very preterm population. Compared with published data from other preterm populations over a shorter review period, the most common causes of death in this population were similar [9, 10]. The most significant difference in this population was a relative higher incidence of major IVH as the principal cause of death [11, 12]. This is an interesting finding as the overall incidence of major IVH in our preterm population is comparable with other preterm populations [13]. As survival rates in extreme prematurity continue to improve, neonatologists are increasingly focused on neurological outcomes in surviving infants. There is an increasing tendency to redirect care in the setting of major IVH within this network, which is very likely to have impacted on cause-specific mortality in the extremely preterm groups. There is likely to be practice variation with respect to redirection of care between NICUs, however, these differences are difficult to objectively assess. Cause-specific mortality was not calculated for each individual unit, which may have allowed analysis of the effects of practice variation on cause-specific mortality.

Death due to ARI was expectedly the leading cause of mortality in the extreme preterm population however infants in older gestational age groups were more likely to die from other causes such as IVH and asphyxia. It is not surprising that perinatal asphyxia becomes the most common cause of death with increasing gestation as the infants in these groups are less likely to die from problems that are more prevalent in the extreme preterm population such as respiratory problems.

As expected, the most significant predictor of death in our preterm population was gestational age regardless of the cause of death. Other perinatal factors that were associated with a higher mortality were male gender, low birth weight, small for gestational age and low Apgar score at five minutes. All of these results are consistent with previously published data [6–8, 14–17].

There were a number of perinatal factors that influenced the cause-specific mortality in this population. Hypertensive disease during pregnancy was associated with a decreased number of deaths due to major IVH. This is consistent with findings from other studies, which show that hypertensive disease is protective for major IVH [18, 19]. Chorioamnionitis was found more prevalently in infants who died from sepsis, which was also consistent with other studies that show an association between maternal chorioamnionitis and neonatal sepsis [19, 20]. It was not surprising to find that infants who die due to respiratory problems, IVH and asphyxia are less likely to have received steroids antenatally. Other expected findings included associations between being outborn and death due to IVH [21] and having no antenatal care and death due to perinatal asphyxia [22].

It is interesting to note that although maternal age was not different among infants who died and infants who survived, the average maternal age varied with the cause of death. Mothers of infants who died due to necrotising enterocolitis had a lower average age, which is consistent with published data from large preterm populations that show that the incidence of necrotising enterocolitis is lower in infants born to older mothers [23]. Mothers of infants who died due to perinatal asphyxia had a higher average age, which is consistent with literature that suggests that advanced maternal age is associated with neonatal encephalopathy in term infants [22].

The period of vulnerability of death varied significantly with each cause of death. Infants who die from ARI and IVH usually die within the first two to three weeks of life, which is an expected result. The majority of deaths relating to sepsis died early reflecting early onset sepsis. The survival curve decreases relatively steadily after this reflecting variability in the onset of late sepsis. Very premature infants continue to be vulnerable to NEC [24, 25] and thus deaths due to NEC behaved very differently to the other common causes of death. There are very few early deaths attributable to NEC followed by a steady decrease in the survival curve due to the onset of NEC after two to three weeks of age.

Analysis of our surrogate markers of resource consumption consistently showed that there are significant differences depending on the cause of death. It is clear that infants who die from ARI and IVH consumed the least resources during their hospital stay. Infants who died from sepsis consumed more resources than infants who died in the immediate postnatal period but less than those who died from NEC. This is unsurprising and is consistent with the mean age at death and survival curves for these causes of death.

This study represents the largest analysis of causes of death in preterm infants, including data from multiple NICUs across a large area. Although analysed retrospectively, all NICUS data are collected prospectively. One of the major strengths of this study compared with previous similar studies is that there was a detailed investigation to ascertain the exact cause of death for every infant. An accurate immediate cause of death was determined and agreed upon for all infants who died during the study period.

The most significant limitation of this study is that to be eligible for inclusion, infants must have been admitted to a NICU. The study provides no information on the foetal mortality associated with important prenatal risk factors, which potentially biases the associations between these risk factors and cause of death. Infants who were born alive at the threshold of viability but not actively resuscitated are also not included in the analysis, which may bias the distribution of cause of death in the extreme preterm population.

It is important to note that the NICUS Data Collection is a regional audit from 10 different NICUs. These data were not separated by NICUS and we were therefore not able to analyse the cause-specific mortality for each unit individually. Although these NICUs have comparable outcomes and all participate in regular meetings to discuss management of the preterm infant, practice variation between units is difficult to assess and may have an effect on cause-specific mortality.

## Conclusions

In performing a detailed analysis of the causes of death in this population, this study provides insight into the specific reasons that preterm infants die and demonstrates that perinatal factors have a significant impact on cause-specific mortality. The varied timing of death, depending on the primary cause of death, highlights that preterm infants who survive the immediate newborn period remain vulnerable to life-threatening conditions such as sepsis and necrotising enterocolitis.

## Abbreviations

ACT: Australian Capital Territory
ARI: Acute repiratory illness
CMR: Cause-specific mortality rate
IVH: Intraventricular haemorrhage
NEC: Necrotising enterocolitis
NICU: Neonatal intensive care unit
NICUS: Neonatal intensive care units’ data collection
NSW: New South Wales
